# Sexual health and serotonin 4 receptor brain binding in unmedicated patients with depression—a NeuroPharm study

**DOI:** 10.1038/s41398-023-02551-x

**Published:** 2023-07-06

**Authors:** Annika Læbo Rasmussen, Søren Vinther Larsen, Brice Ozenne, Kristin Köhler-Forsberg, Dea Siggaard Stenbæk, Martin Balslev Jørgensen, Annamaria Giraldi, Vibe G. Frokjaer

**Affiliations:** 1 Neurobiology Research Unit, Rigshospitalet, Copenhagen, Denmark; 2 Department of Clinical Medicine, Faculty of Health and Medical Sciences, University of Copenhagen, Copenhagen, Denmark; 3 Department of Public Health, Section of Biostatistics, University of Copenhagen, Copenhagen, Denmark; 4 Psychiatric Centre Copenhagen, Mental Health Services Capital Region of Denmark, Copenhagen, Denmark; 5 Department of Psychology, University of Copenhagen, Copenhagen, Denmark; 6 Sexological Clinic, Mental Health Services Capital Region of Denmark, Copenhagen, Denmark

**Keywords:** Depression, Molecular neuroscience

## Abstract

Sexual dysfunction is prominent in Major Depressive Disorder (MDD) and affects women with depression more than men. Patients with MDD relative to healthy controls have lower brain levels of the serotonin 4 receptor (5-HT_4_R), which is expressed with high density in the striatum, i.e. a key hub of the reward system. Reduced sexual desire is putatively related to disturbed reward processing and may index anhedonia in MDD. Here, we aim to illuminate plausible underlying neurobiology of sexual dysfunction in unmedicated patients with MDD. We map associations between 5-HT_4_R binding, as imaged with [^11^C]SB207145 PET, in the striatum, and self-reported sexual function. We also evaluate if pre-treatment sexual desire score predicts 8-week treatment outcome in women. From the NeuroPharm study, we include 85 untreated MDD patients (71% women) who underwent eight weeks of antidepressant drug treatment. In the mixed sex group, we find no difference in 5-HT_4_R binding between patients with sexual dysfunction vs normal sexual function. However, in women we find lower 5-HT_4_R binding in the sexual dysfunctional group compared to women with normal sexual function (β = −0.36, 95%CI[−0.62:−0.09], *p* = 0.009) as well as a positive association between sexual desire and 5-HT_4_R binding (β = 0.07, 95%CI [0.02:0.13], *p* = 0.012). Sexual desire at baseline do not predict treatment outcome (ROC curve AUC = 52%[36%:67%]) in women. Taken together, we find evidence for a positive association between sexual desire and striatal 5-HT_4_R availability in women with depression. Interestingly, this raises the question if direct 5-HT_4_R agonism can target reduced sexual desire or anhedonia in MDD.

## Introduction

Sexual dysfunction (e.g., low sexual desire, arousal difficulties and anorgasmia) is a prominent feature of major depressive disorder (MDD) reaching a prevalence as high as 75% [1], and often leads to a decline in quality of life [2]. Indeed, 42% of male and 50% of female patients with untreated MDD experienced a decreased sexual drive [3]. Sexual dysfunction more often affects women with depression compared to men [3, 4]. Interestingly, a review from 2018 concluded that the greatest risk factor for sexual dysfunction in women is depression itself and that a particularly strong link between depression and low sexual desire is evident [5]. Sexual desire specifically refers to a person’s interest in sexual objects or activities and motivation for sex and/or sexual fantasies. Reduced sexual desire might reflect overall perceived anhedonia (i.e., lack of interest in what is usually pleasurable), which is a core symptom of MDD. In line with this, a study linked anhedonia in women with depression to lower sexual desire [6]. Anhedonia is putatively linked to disturbances in reward circuit brain functioning [7]. Even though dopamine is traditionally thought of as the key neurotransmitter for reward processing, serotonin also modulates reward circuit function especially when encoding social reward [8, 9]. Therefore, intriguingly, serotonin signalling may critically modulate the encoding of social reward, including sexual reward, and thus possibly affect sexual motivation and desire as indirectly supported by others [10, 11]. Interestingly, reward brain circuit function appears to differ substantially between men and women, which has been shown in a recent study of brain responses to monetary reward; relative to women, men displayed more intense recruitment of the reward circuit in response to high vs low salience stimuli [12]. Therefore, the neurobiological basis of anhedonia and disturbed sexual desire in depression may differ with sex such that serotonergic influence on reward system functioning and sexual health is particularly pronounced in women with MDD.

The serotonin 4 receptor (5-HT_4_R) has been proposed as a promising antidepressant target [13, 14]. Preclinical data support the involvement of the 5-HT_4_R in treatment mechanisms of anxiety and depression; for instance, administration of 5-HT_4_R agonists to rodents generates rapid anxiolytic-like and antidepressant-like behavior [15, 16], possibly via supporting hippocampal neurogenesis [17]. Further, novel work from our group shows that patients with MDD have significantly lower cerebral 5-HT_4_R binding than healthy controls [18]. In humans, striatal 5-HT_4_R binding is lower in healthy individuals with familial risk of mood disorders [19], which may represent a trait risk marker for depression. Further, healthy women appear to have significantly lower levels of the receptor in the brain when compared to men [19]. The 5-HT_4_R is highly expressed in central hubs of the reward circuit (e.g., the striatum) [20, 21] and striatal 5-HT_4_R binding potential has been shown to be positively correlated to ventral striatum activity in relation to monetary reward in healthy women [22]. Based on the anatomical expression of the receptor with high densities in a key hub in the reward system, the 5-HT_4_R could be functionally important for modulating reward processing and anhedonia in MDD, which may underlie the ability to experience sexual desire, even though until now no human (or rodent) data have been available to shed light on such a phenomenon. Nevertheless, indirect evidence suggests that 5-HT_4_R signalling may be related to reward system related functions in terms of appetite and eating behaviour; 5-HT_4_R knock out mice exhibit hypophagia when stressed and re-expression of 5-HT_4_R in the medial prefrontal cortex rescues this phenotype [23].

Sexual dysfunction is not only key in depression symptomatology; although the literature is ambiguous, loss of sexual desire and other sexual side effects are listed as potential side effects to antidepressant treatments targeting the serotonin system. The neurobiology of desire is believed to be an interplay between excitatory and inhibitory systems, where serotonin constitutes a key inhibitory neuromodulator, since it suppresses the ability of excitatory systems (e.g. the dopamine system) to be activated by sexual cues [24]. In a group of 704 MDD patients treated with an antidepressant drug (selective serotonin reuptake inhibitor, SSRI or serotonin-norepinephrine reuptake inhibitor, SNRI), around 50% of the pooled cohort of men and women developed or worsened the existing state of low sexual desire [25]. On the contrary, other studies found that sexual function in women (including sexual desire) improves during antidepressant treatment in parallel with the remission of depressive symptoms, while the opposite may be true for men [26, 27]. Improvement of sexual functioning across 12 weeks of antidepressant treatment including SSRIs has already been observed in the present study cohort, although we observed no sex-difference [28]. Nevertheless, within women there might be differences in SSRI treatment efficacy which depends on reproductive states and use of hormonal contraception since the use of hormonal contraception affects both sexual desire [29] and brain levels of 5-HT_4_R [30].

The overall aim of this study is to illuminate the underlying brain biology of sexual dysfunction in the depressed state and evaluate if sexual dysfunction can help predict antidepressant treatment outcomes. More specifically, the objectives are (1) To investigate associations between 5-HT_4_R binding in the striatum and self-reported sexual function in the depressed state, (2) To determine if striatal 5-HT_4_R binding is specifically related to sexual desire and (3) In an exploratory analysis, in women only, to map if sexual desire can predict treatment outcome in depression after eight weeks of treatment, since a main scope of the NeuroPharm trial was to evaluate potential markers of treatment response.

We hypothesise that sexual function, in particular sexual desire in women, is positively associated with striatal 5-HT_4_R binding.

## Materials and methods

### Participants

We included patient data from the NeuroPharm clinical trial, which investigated a set of prognostic biomarkers for recovery following standard treatment for depression in a naturalistic setting (ClinicalTrials.gov, identifier: NCT02869035) [31]. All participants gave written informed consent, and the study was approved by the Committees on Health Research Ethics in the Capital Region of Denmark (Protocol ID: H-15017713). Data were available from the Center for Integrated Molecular Brain Imaging (Cimbi) database [32]. Upon inclusion, patients were required to meet the DSM-5 criteria for single or recurrent unipolar depression and to rate more than 17 points on the 17 item Hamilton Depression Rating Scale (HDRS-17) [33]. Some highlighted exclusion criteria were a) use of pharmacological antidepressant treatment within two months prior to enrolment, b) more than one attempt with an antidepressant treatment in the current episode, c) use of medical treatment affecting the central nervous system (e.g. metoclopramide, serotonergic migraine medicine) and d) acute suicidal ideation or psychosis. The complete list can be found in the study protocol [31]. At baseline, the study cohort was positron emission tomography (PET)-scanned and self-reported information about sexual function was collected. Afterwards, patients started treatment with escitalopram (SSRI) in a flexible dose from 5–20 mg and had the option to switch to duloxetine (SNRI) after four weeks if needed. Information on sexual function was recollected after 8 weeks of antidepressant treatment. Of the pre-registered study population of 100 patients, 92 patients were scanned at baseline. The reasons for not scanning were: Acute suicidal ideation (*n* = 1); tracer production failure (*n* = 1), excessive anxiety not compatible with scanning procedure (*n* = 2), unexpected pregnancy discovered at site (*n* = 2); withdrawal of consent (preferred psychotherapy) (*n* = 2). From the original 92 patients, seven patients were excluded in this study due to; missing data on sexual function (*n* = 2), spontaneous remission before baseline assessment (*n* = 1), injected tracer mass dose exceeding the suggested dose of 4.5 μg [34] (*n* = 1), interrupted PET-scan (*n* = 1) and women over 50 years of age (*n* = 2), since we cannot exclude that hormonal changes related to the perimenopause and menopause will influence 5-HT_4_R binding [30] and sexual function.

### Questionnaires

#### Changes in Sexual Functioning Questionnaire

Sexual function was quantified using the validated Danish version of 14-item Changes in Sexual Functioning Questionnaire (CSFQ-14) [35] which was completed online in a private setting. The CSFQ-14 is validated in both healthy and depressed cohorts and is available in a male (CSFQ-14-M) and a female (CSFQ-14-F) version [36]. Each item is scored from one to five points and the sum gives a total score between 14 and 70 with a validated clinical threshold of 41 for women and 47 for men [36], where a score less than or equal to the threshold indicates sexual dysfunction. We here refer to sub-threshold patients as “sexually dysfunctional”, although we acknowledge the fact that “sexually dysfunctional” is a clinical diagnosis that cannot be directly deduced from the CSFQ-14. Five dimensions can be extracted from the CSFQ-14: Pleasure, desire/frequency, desire/interest, arousal/excitement and orgasm/completion. Additionally, we asked the question “How important is it to you to have a good sex life?”. Here, importance is scored from one to four points, where one indicates the greatest importance.

#### The Snaith Hamilton Pleasure Scale

The Snaith Hamilton Pleasure Scale (SHAPS) [37] is a 14-item self-administered tool for assessment of hedonic tone and quantifies an individual’s ability to experience pleasure, enjoyment and satisfaction within the last days. The sum yields a total score with a range from zero to 14, where a higher score indicates a greater degree of anhedonia.

#### The Hamilton Depression Rating Scale

We used the HDRS-17 to characterise depression severity at baseline and after eight weeks of treatment. We applied the relative change in Hamilton Depression Rating Scale 6 items in percentage from baseline to week eight (rΔHDRS-6) as a measure of clinical recovery, which was used in longitudinal analyses. For prediction analyses we dichotomised the recovery measure, as either “good” (50% or more reduction in HDRS-6 following eight weeks of antidepressant treatment (HDRS_50%_) or “bad” (the rest) [38].

### Neuroimaging

PET neuroimaging was obtained using a high-resolution research tomography Siemens PET-scanner (CTI/Siemens, Knoxville, TN, USA). Participants underwent a 6 min transmission scan for correction purposes. Following this, an intravenous bolus of approximately 600 MBq of the PET tracer ligand [^11^C]SB207145 was administered over 20 s succeeded by a 120-min dynamic PET data acquisition [31]. MRI (Magnetic Resonance Imaging) scans were acquired using a Siemens 3-Tesla Prisma scanner (Erlangen, Germany). The high-resolution structural T1 weighted images were segmented into grey matter, white matter and cerebrospinal fluid and aligned and co-registered to PET images using Statistical Parametric Mapping (SPM8). PET scans were motion corrected using Air 5.2.5 [39]. The pallidostriatum was selected as the region of interest since striatal density of 5-HT_4_Rs is high and the striatum is a key hub in the reward circuity [21, 40]. The Pvelab software package was used to extract the region of interest [41]. To estimate the non-displaceable binding potential (BP_ND_) of the 5-HT_4_R, mean tissue time activity curves for grey matter volumes were extracted for kinetic modelling, where the simplified reference tissue model (SRTM) was used. Here, the cerebellum (excluding vermis) was used as a reference region since it has a negligible 5-HT_4_R specific binding [21]. BP_ND_ is proportional to the density of 5-HT_4_R or the number of receptors available for binding. In this paper, “binding” is used synonymously with BP_ND_.

### Statistics

Demographics and clinical profiles at baseline (proportions, means and standard deviations) were compared between patients with sexual dysfunction vs. patients with normal sexual function (defined by the CSFQ-14-F/M total score thresholds). *P*-values were calculated using multiple regression models, Welch’s two-sample t-test for continuous variables, Fischer’s exact test for binary variables, and Mann Whitney U test for the ordinal variable “how important is it to you to have a good sex life?”. Analysis of variance (ANOVA) and Tukey’s Honest Significant Difference post hoc test (corrected for multiple comparisons, i.e. three) were used to compare sexual desire/interest scores between hormonal contraceptive user status groups (i.e. oral contraceptive users, hormonal IUD users and non-users) in a women-only analysis. 95% confidence intervals (CI) are reported, and p-values below 0.05 were considered statistically significant. Code is available upon request.

Although the NeuroPharm clinical trial intended to evaluate sexual dysfunction in the depressed state, sex-specific analyses were not pre-registered. We disclose that the following are exploratory, yet hypothesis-driven, unplanned analyses.

#### Pre-treatment analyses

To address objective (1), we compared baseline 5-HT_4_R binding between “sexually dysfunctional” patients and patients with “normal sexual function” based on the CSFQ-14-F/M total score thresholds using multiple linear regression models. Then, we tested if the difference in 5-HT_4_R binding between the two groups (“sexually dysfunctional” vs. “normal sexual function”) was dependent on sex in a sexual function BY sex interaction analysis.

Further, we tested associations between 5-HT_4_R binding and the CSFQ-14 total scores on a continuous scale. As the scales for men and women are not identical, this was conducted in each sex, separately, using multiple linear regression models.

To address objective (2), we tested a potential association between CFSQ-14’s desire/interest dimension and striatal 5-HT_4_R binding using multiple linear regression. Using the same method, we also tested associations between the remaining CSFQ-14 dimensions and striatal 5-HT_4_R binding as explorative analyses.

In (1) and (2) we only report estimates of the women in the main manuscript due to low power in the men-only group (see supplementary).

In an exploratory analysis, we used linear regression models (unadjusted for covariates) to evaluate the association between anhedonia scores (SHAPS total score) and sexual desire/interest in women.

#### Longitudinal analyses (women only)

In objective (3), we wanted to illuminate if the level of sexual desire in the depressed state could be used as a proxy for underlying serotonin brain biology and thus as a tool to predict treatment outcomes in depression. Therefore, we tested if baseline sexual desire score predicted HDRS_50%_ at week eight by using the area under the ROC curve (AUC), where an AUC of 0.5 indicated a predictive value of the model equal to chance and an AUC of 1.0 a perfect predictive value of 100%.

To take the potential contribution of hormonal contraceptive use into account, we also assessed the predictive value of the sexual desire/interest score combined with hormonal contraceptive user status in an analysis adjusting for age. For this, we estimated the area under the ROC curve (AUC) using repeated 10-fold cross-validation (100 repetitions).

#### Model considerations

The multiple linear regression models were adjusted for the following co-variates as they are considered to influence 5-HT_4_R binding: (1) Age, (2) injected [11 C]SB207145 mass per kg bodyweight, (3) 5-HTTLPR status (LALA/non-LALA) [42] in addition to (4) sex [43] in the analysis that include both men and women.

Further, we used depression severity (HDRS-16 item) and oral hormonal contraception use (in women only) as covariates in sensitivity analyses for the estimated effects, since hormonal contraception has been linked to both female sexual dysfunction [44] and development of depressive episodes [45]. The influence of oral hormonal contraception use on the above mentioned analyses was tested using oral hormonal contraception use as a covariate in a subgroup of women (*n* = 50), who either used oral contraceptives (*n* = 25) or no hormonal contraception (*n* = 25).

## Results

### Study population

Table 1 shows demographical, psychometrical and hormonal profiles as well as PET parameters of the study population (*n* = 85). Moreover, it compares and displays how the parameters are distributed within the group of patients with sexual dysfunction (*n* = 54) and the group of patients with normal sexual function (*n* = 31) (defined by the CSFQ-14-F/M total score thresholds).

**Table 1:** Demographical and clinical profile at baseline assessment.

Clinical parameters	Total	Normal sexual function	Sexually dysfunctional	*p*-value
		CSFQ-14 total score > 41 for women/>47 for men	CSFQ-14 total score ≤41 for women/≤47 for men	*G*roup with normal sexual function vs sexually dysfunctional
	*n* = *85*	*n* = 31	*n* = 54	
*Sex*				
Female ♀	60 (70.6%)	14 (45.2%)	46 (85.2%)	0.00017*
Age (years)	26.1 ± 6.4	26.2 ± 5.7	26.1 ± 6.8	0.94
BMI (kg/m^2^)	24.5 ± 5.7	23.7 ± 4.7	24.9 ± 6.2	0.36
*5-HTTLPR status*				
LA/LA	23 (27.1%)	6 (19.4%)	17 (31.5%)	0.31
HDRS-17 score	22.7 ± 3.4	21.7 ± 3.1	23.3 ± 3.5	0.039*
HDRS-16 score (without sexual item)	21.6 ± 3.3	21.9 ± 3.3	21.4 ± 3.4	0.57
HDRS-6 score	12.3 ± 1.7	12.1 ± 1.5	12.4 ± 1.8	0.44
SHAPS score	7.7 ± 3.2	6.5 ± 3.3	8.4 ± 3	0.005*
♀ *Hormonal contraception use*				
OC	41.66%	21.4%	47.83%	0.12
	(25/60 women)	(3/14 women)	(22/46 women)	
H-IUD	16.66%	42.9%	8.70%	0.007*
	(10/60 women)	(6/14 women)	(4/46 women)	
Non-hormonal	41.66%	35.7%	43.48%	0.76
	(25/60 women)	(5/14 women)	(20/46 women)	
*PET parameters*				
SB injected dose (MBq)	578.8 ± 56.2	577.3 ± 51.3	579.7 ± 59.3	0.85
SB injected mass (µg)	0.9 ± 0.9	0.9 ± 1	0.9 ± 0.8	0.81
SB injected mass per body weight (µg/kg)	0.013 ± 0.013	0.013 ± 0.015	0.013 ± 0.012	0.87
SB SA in cerebellum (GBq/µmol)	352.6 ± 186	368.5 ± 212	343.5 ± 170.8	0.55
*Sexological data*				
CSFQ-14 total score	39.9 ± 10.3	50.8 ± 6.7	33.6 ± 5.6	
“How important is it for you to have a good sex life?”	Very important = 22 (26%)	Very important = 13 (42%)	Very important = 9 (17%)	0.008*
	Important = 38 (45%)	Important = 12 (39%)	Important = 26 (48%)	
	Not so important = 14 (16%)	Not so important = 4 (13%)	Not so important = 10 (19%)	
	Not at all important = 5 (6%)	Not at all important = 0 (0%)	Not at all important = 5 (9%)	
	Don’t know = 6 (7%)	Don’t know = 2 (6%)	Don’t know = 4 (7%)	

Mean-values with standard deviations and frequencies and percentage are displayed when relevant, *indicates *p*-values below 0.05. The statistical tests performed were two-sided *t* test for the continuous variables and Fischer’s exact test for binary variables and Mann–Whitney *U* test the for ordinal variable (“How important is it for you to have a good sex life?”). Hamilton-16 score: The Hamilton-17 total score minus the score of the sexual item (item 14) of the Hamilton scale. 5-HTTLPR status: The status of the serotonin transporter gene linked polymorphic region coded as a binary variable (LA/LA vs. non-LA/LA).

*OC* oral contraception, *H-IUD* hormonal intrauterine device, *non-hormonal* non-hormonal contraception/non-user.

### Pre-treatment

#### Sexual health, anhedonia measures and role of hormonal contracepti on within women with depression

According to the clinical CSFQ-14 threshold, 54 patients (64% of the patients in total) met the criteria for being sexually dysfunctional at baseline. Notably, a greater proportion of these patients were women (77% of the women vs. 32% of the men, *p* = 0.00017). The age range was 18.6–43.2 years for women and 18.3–48.9 years for men. Patients who belonged to the sexually dysfunctional group also perceived more overall anhedonia (mean SHAPS 8.4 vs. 6.5, *p* = 0.005) and considered having a good sex life less important (*p* = 0.008). 81% of the patients with normal sexual function considered having a good sex life “important” or “very important”, whereas the same was only true for 65% of the patients with sexual dysfunction. Moreover, a group difference was observed in depressive symptoms rated on the HDRS-17 (*p* = 0.039), but not on the HDRS-6 scale (*p* = 0.44) or on the HDRS-16 scale (*p* = 0.57), where we excluded the item assessing sexual function.

Although the group of women in the depressed state with normal sexual function was small (*n* = 14), we observed that a larger proportion of women with normal sexual function used hormonal intrauterine devices (IUD) compared the group of women with sexual dysfunction (43% vs. 9%, *p* = 0.007). No statistical difference was found in the proportions of oral contraceptive users between the groups. However, desire/interest scores appeared to differ between the three hormonal contraceptive groups (i.e., oral contraception users, IUD users and non-users) (ANOVA, *p* = 0.01). The post hoc analysis showed that this was driven by lower sexual desire in oral hormonal contraceptive users compared to hormonal IUD users (β = −2.1, 95%CI [−3.8;−0.3], p.adj = 0.02) and non-users (β = −1.4, 95%CI [−2.7;−0.4], p.adj = 0.04).

### Association between sexual function and 5-HT_4_R binding

In the following, we compared 5-HT_4_R binding between patients with depression with and without sexual dysfunction. First, this was done in a mixed group of men and women and next in sex-specific analyses (e.g. women-only and men-only). Here, of the 60 women in total, 46 met the criteria for sexual dysfunction and 14 did not. Of the 25 men in total, 8 met the criteria and 17 did not.

**Fig. 1:**
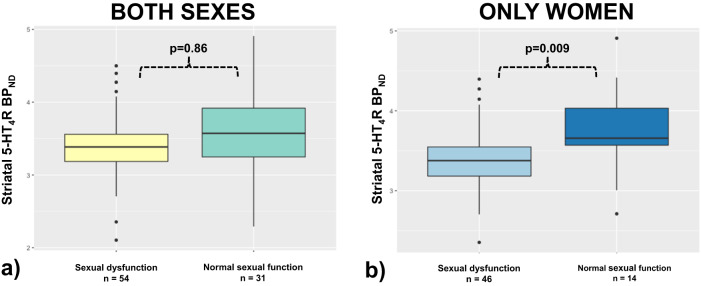
Sexual function and striatal 5-HT_4_R binding. **a** Difference in 5-HT_4_R binding potential between sexually dysfunctional patients (both men and women) with depression and those with normal sexual function (β = 0.05, *p* = 0.86). **b** Women separately. Difference in 5-HT_4_R binding potential between sexually dysfunctional and those with normal sexual function (β = −0.36, *p* = 0.009). Sexual dysfunction was defined as CSFQ-14 total scores above or below the clinical threshold (in women defined as a CSFQ-14 total score ≤ 41, in men ≤47).

When we compared baseline striatal 5-HT_4_R binding between patients with depression (both men and women) with normal sexual function to those with sexual dysfunction, we found no evidence of a difference (β = 0.04, 95%CI [−0.38:0.45], *p* = 0.86, Fig. 1a). In an interaction analysis, we did not find substantial evidence to support that a difference in 5-HT_4_R BP_ND_ between depressed patients with normal sexual function and sexual dysfunction was sex-dependent (β = −0.40, 95%CI [−0.91:0.11], *p* = 0.13). However, we pursued to compare 5-HT_4_R binding in a women-only subgroup since the interaction analysis was somewhat underpowered. In the women-only subgroup, we observed a difference in 5-HT_4_R binding (sexual dysfunctional group vs group with normal sexual function) (β = −0.36, 95%CI [−0.62:−0.09], *p* = 0.009, Fig. 1b). We did not consider it meaningful to interpret estimates in the men-only subgroup due to low power (see supplementary).

We found no evidence of an association between baseline CSFQ-14 total score and baseline striatal 5-HT_4_R binding in women (β = 0.010, 95%CI [−0.006:0.026], *p* = 0.20). We replicated the analysis adjusting for depression severity (HDRS-16) and oral hormonal contraception use and found similar results. Again, due to low power in the men-only group, we did not find it meaningful to interpret the association in men only analyses; however, the estimates are provided in supplementary material.

### Association between sexual desire and 5-HT_4_R binding

**Fig. 2:**
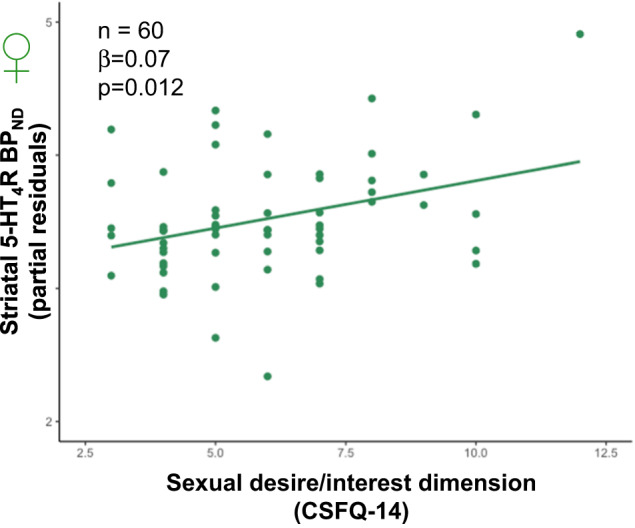
Sexual desire/interest dimension (CSFQ-14) and striatal 5-HT_4_R binding in women. The positive association between the sexual desire/interest dimension of CSFQ-14 and 5-HT_4_R binding potential in striatum in women at baseline. β = 0.07, p_adj_ = 0.024. The 5-HT_4_R binding displayed on the graph are the original values corrected for age, injected [11 C]SB207145 mass per kg bodyweight and 5-HTTLPR status.

We found a positive association between baseline scores of the CSFQ-14 dimension sexual desire/interest and baseline striatal 5-HT_4_R binding in women (β = 0.07, 95%CI [0.02:0.13], *p* = 0.012, Fig. 2), i.e., women scoring lower on the desire/interest dimension of the CSFQ-14 also had lower 5-HT_4_R binding in the striatum. Once again, we replicated the analysis adjusting for depression severity (HDRS-16) and oral hormonal contraception use and found similar results.

In explorative analyses, we tested if associations existed between the remaining dimensions of the CSFQ-14 and striatal 5-HT_4_R binding in women in order to evaluate potential contributions from other dimensions than desire/interest. We found no such associations in any of the remaining four dimensions (see Table 2). See supplementary material for a full overview of the estimates of the associations between the CSFQ-14 dimensions and striatal 5-HT_4_R binding in each sex.

**Table 2:** Dimensions of the CSFQ-14 in women.

	Women	
Dimensions	Estimate, 95% CI	*p*-value
Pleasure	*β* = 0.07, [−0.05:0.20]	0.25
Desire/frequency	*β* = −0.006, [−0.09:0.07]	0.88
Desire/interest	*β* = 0.07, [0.02:0.13]	0.012*
Arousal	*β* = 0.01, [−0.05:0.06]	0.72
Orgasm	*β* = 0.02, [−0.03:0.07]	0.51
Total score	*β* = 0.010, [−0.006:0.026]	0.20

Associations between 5-HT_4_R binding potential in the striatum and the different dimensions of CSFQ-14.

95% CI = 95% confidence interval. All *p*-values are unadjusted.

### Anhedonia, sexual desire and 5-HT_4_R binding

In women we found a negative association between anhedonia scores (SHAPS total score) and the CSFQ-14 dimension concerning sexual desire/interest (β = −0.48, 95%CI [−0.85:−0.11], *p* = 0.013). Here, more overall anhedonia meant lower sexual desire/interest scores. We adjusted for depression severity (HDRS-16) and oral hormonal contraception and found similar results.

To evaluate if the observed association between sexual desire and 5-HT_4_R binding in women was confounded by overall perceived anhedonia, we also adjusted for the SHAPS total score in a sensitivity analysis. However, this did not change the association substantially (β = 0.06, 95%CI [0.004:0.12], *p* = 0.036) and it revealed no association between the SHAPS score and 5-HT_4_R binding in women (*p* = 0.32).

### Longitudinal facet of the study

During the eight weeks of pharmacological antidepressant treatment, the women on average increased their sexual desire/interest scores by 17% (min: −58%, max: 100%).

Pre-treatment sexual desire/interest scores did not show predictive power to distinguish a 50% or more reduction in depressive symptoms (HDRS_50%_). Here, the AUC was 51.7% (95% CI [36%:67%]), which was not statistically significantly above chance, and this did not change when hormonal contraceptive user status was added to the model (AUC = 44.4%).

## Discussion

In the total patient group of unmedicated men and women with depression, we found no evidence of a difference in 5-HT_4_R binding between those with normal sexual function and those with sexual dysfunction. However, in women only we observed lower binding in individuals with sexual dysfunction compared to individuals with normal sexual function. This appeared to be driven by a positive association between sexual desire/interest and striatal 5-HT_4_R binding in women. We found no evidence supporting that pre-treatment sexual desire/interest was predictive of recovery following eight weeks of antidepressant treatment in women.

### Sexual dysfunction and reward system serotonergic dysfunction in MDD

The observed difference in 5-HT_4_R binding within women with sexual dysfunction and normal sexual function in addition to the coupling between low 5-HT_4_R binding and poorer sexual desire supports the hypothesis of low 5-HT_4_R agonism capacity in depression [46]. The 5-HT_4_R is anatomically expressed in abundance in the reward system (i.e. the striatum) [20, 21] and if the receptor is less active and/or less available for binding the result could be less downstream actions, which in turn could compromise reward system functioning, as also supported by earlier work from our group [22]. To the best of our knowledge, no research has been published about the link between sexual function and the 5-HT_4_R. However, evidence from both rodent and human studies suggests the 5-HT_4_R plays an important role in hunger and eating [23]—another basic motivation-driven behaviour that inhabits the reward circuitry. Indeed, a review article concluded that the functional neuroanatomy of sexual pleasure resembles that of other pleasures [47]. This reconciles with the notion that low 5-HT_4_R agonism capacity may be coupled to reduced reward-seeking behaviour. Other studies by our group find that the weight loss following gastric bypass surgery in obese humans is modulated by serotonin neurotransmission [48] and that obese humans have higher levels of 5-HT_4_R in the reward system [49].

In the present data, we see evidence for a positive association between 5-HT_4_R binding and sexual desire/interest in women, indicating a role of 5-HT_4_R direct agonism capacity on sexual desire/interest—at least for women.

In women, we find an association between overall anhedonia scores and sexual desire indicating that sexual desire, or lack hereof, appears to be more clearly linked to anhedonia in women with depression. The internal validation of the CSFQ-14 argues that desire/interest differs substantially from desire/frequency, pleasure and arousal as they cluster in different factor patterns [35]. Sexual desire may very well reflect the reward system more directly than other dimensions of sexual health, which is in line with the widely accepted incentive-based sex response cycle theory [50]. In this theory, sexual desire is not necessarily innate but can also be triggered or excited as a response to arousal and is described in an interplay of somatic, psychosocial and neurobiological factors. Motivation is important and it has been proposed that a potential vicious cycle can arise in the depressed state where anhedonia diminishes the wanting of physical pleasure and emotional intimacy which leads to diminished sexual incentive [5]. Especially women may be more vulnerable to decreases in motivation. A study on a Danish population of men and women showed that women more often had sex due to a motivation to experience desire or the wish for intimacy, compared to men who more often reported arousal to be the reason [51]. A study conducted in healthy women found that anhedonia was coupled to poorer same-day sexual desire further supporting this theory [52], which also aligns with our data. Intriguingly, we found that sexual desire/interest was coupled to 5-HT_4_R independent of the level of broader measures of anhedonia for the women with depression. We speculate that sexual desire/interest yields a more direct measure of hedonic tone in the reward system than overall anhedonia and may prove to be an important tool in the clinical assessment – perhaps in particular for women with depression. Reconciling with this notion, the drug Flibanserin, a combined 5-HT_1a_ agonist and 5-HT_2_ antagonist, is used to treat low desire in women, once again indicating that modulation of the serotonergic system is important for the level of perceived sexual desire [53].

### Sex differences in sexual function in the depressed state

In line with previous studies, women in our study more frequently met the threshold for being sexually dysfunctional than men [3, 4]. Regarding sexual desire, several studies point to sex differences too: A Danish study found that amongst the general population men have significantly higher desire than women [54]. In the depressed state, gender differences are less well elucidated. Some evidence states that women with depression most often are negatively influenced by low desire [55]. Another study found that sexually dysfunctional men and women experience the same emotions (i.e. significantly less pleasure and satisfaction, and more sadness, disillusion, guilt, and anger) when compared to sexually functional [56].

Although sexual desire/interest could not predict treatment outcome in our female subgroup, a previous study indicates that the effect of antidepressant treatment might be more hung up on amelioration of anhedonic symptoms for women than for men. That study found that female sex predicted remission of anhedonia (i.e., here defined as a SHAPS total score <3 after eight weeks of vortioxetine treatment) [57].

We are underpowered to determine effects in the male group (25 men vs 60 women). Therefore, we cannot exclude a similar pattern in the male cohort as well as we cannot exclude a potentially lower effect size in men or a different pattern of associations between dimensions of the CSFQ-14 and 5-HT_4_R binding. The interaction analysis of “sex BY sexual function status” was statistically insignificant but also somewhat underpowered. Therefore, we cannot firmly exclude that it is irrelevant to perform the analyses independent of sex. Accordingly, we disclose both the mixed-sex analyses, interaction analysis and sex-specific analyses (men-only analyses in supplementary) to cover all available data.

### Contraception use

Although we observe a difference in the distribution of contraception use between the women with and without sexual dysfunction, we interpret that with caution since the group sizes we compare are small. Nevertheless, oral hormonal contraception users rated significantly lower on the sexual desire/interest item than both hormonal IUD users and non-hormonal contraception users, which highlights the clinical important question: Does use of oral hormonal contraception reduce sexual desire and interest even in the depressed state? Interestingly, some studies link hormonal contraception use in the healthy state to compromised sexual function [44], especially deterioration in sexual desire [29]. Further, the use of hormonal contraception has been linked to the development of depressive episodes [45], where specifically oral hormonal contraception has been suggested to modulate the serotonergic brain signalling in women [30, 58]. Taken together, serotonin receptor 4 may be involved in the mechanisms by which the use of hormonal contraception increase the risk for depression and/or sexual dysfunction in both the healthy and depressed state and might be a critical target in the treatment of depression and sexual dysfunction in women.

### Clinical implications

As MDD is a heterogenous disorder perhaps it is not surprising that one single feature of the symptom complex is predictive of a treatment (SSRI), which is also not specific to this symptom, i.e. sexual desire/interest. Our study highlights the importance of acknowledging sexual health of patients with depression; even in the depressed state (with negative cognitive bias) more than two thirds of the patients in this study found it important or very important to have a good sex life. Consequently, in order to implement a patient-centred approach to treatment of MDD, sexual function should be directly addressed and targeted.

The lower striatal density of 5-HT_4_R in women with low desire might reflect low agonism capacity of signalling via 5-HT_4_R and constitutes a potential target for augmentation of antidepressant treatment [14]. Prucalopride, a 5-HT_4_R agonist, has recently been approved for use in humans and shows pro-cognitive effects [59], even though so far effects on hedonic features in humans have not yet been studied. Work in rodents have shown promising antidepressant, anxiolytic and pro-cognitive results of direct agonism of the 5-HT_4_R [15, 16]. We propose that treatment with 5-HT_4_R agonists may have the potential to improve sexual function and overall anhedonia in MDD, maybe in particular for women, although further research is needed to investigate this.

### Methodological considerations and limitations

Some limitations should be considered: (1) Within the PET field, the sample size used in this study is relatively large. However, with regard to the analyses and comparisons between the smaller subgroups, interpretation should be made with caution, (2) We lack detailed information about the patients’ sexual partner status. Therefore, we cannot consider how partner status would affect our findings, (3) Since the CSFQ-14 has sex-specific threshold values, we could not meaningfully directly compare men and women (unless they were stratified based on the threshold), (4) Clearly, our findings are not exhaustive, meaning that the 5-HT_4_R only explains a small proportion of the variance in sexual phenotype in MDD.

In conclusion, we here demonstrate that in unmedicated women with depression, low sexual desire is associated with lower 5-HT_4_R binding in the striatum, a central hub of the reward circuit. We also show that unmedicated women with depression have lower striatal 5-HT_4_R binding than those with normal sexual function. Our data do not allow conclusive analyses in men with depression. We speculate that our findings may reflect that sexual desire is partly dependent on 5-HT_4_R agonism capacity in the reward brain circuit—at least in women—and thus may be an interesting molecular brain marker or an antidepressant target to investigate in future studies.

## Supplementary information

Supplementary

Supplementary figure 1

Supplementary table 1

## Data Availability

The dataset used in this study is available from the Cimbi database. All researchers can request access to data from the Cimbi database (www.cimbi.dk/db).
