# Supplementary material for: Sexual health and serotonin 4 receptor brain binding in unmedicated patients with depression—a NeuroPharm study
Source: Transl Psychiatry. 2023 Jul 6;13:247. doi: 10.1038/s41398-023-02551-x (PMC10325956; doi:10.1038/s41398-023-02551-x)
Supplement: Supplementary file 1 — Supplementary [file 41398_2023_2551_MOESM1_ESM.docx]

Supplementary – Estimates in the male subgroup

We did not observe a difference in 5-HT_4_R brain binding when we compared the sexual dysfunctional group (n=8) to the group with normal sexual function (n=17) (β=-0.012, 95%CI [-0.58:0.55], p.adj=1, **supplementary figure 1**). Also, we found no evidence of an association between baseline CSFQ-14 total score and baseline striatal 5-HT_4_R binding (β=0.00075, 95%CI [-0.029:0.030], p=0.96). Moreover, no significant association was found between the CSFQ-14 dimension sexual desire/interest and 5- striatal 5-HT_4_R binding in the male patients (β=0.05, 95%CI [-0.05:0.15], p.adj=0.62). A full overview of the estimates of the associations between the CSFQ-14 dimension and striatal 5-HT4R binding in men and women can be found in **supplementary table 1.**

Multiple comparisons in the sex-stratified analyses (i.e., two) were accounted for using the Bonferroni correction.

**SUPPLEMENTARY TABLE 1 – Dimensions of the CSFQ-14 in both men and women**

***Supplementary table 1:*** *Associations between 5-HT_4_R binding potential in the striatum and the different dimensions of CSFQ-14. 95% CI = 95% confidence interval. All p-values are unadjusted.*

**SUPPLEMENTARY FIGURE 1 - Sexual function and striatal 5-HT_4_R binding in both men and women**

***Supplementary figure 1:*** *Women are displayed in blue boxes, men in green. Difference in 5-HT_4_R binding potential between sexually dysfunctional patients with depression and those with normal sexual function - stratified on sex (β_women_=-0.36, p.adj._women_=0.018, β_men_=-0.012, p.adj,_men_=1). Sexual dysfunction was defined as CSFQ-14 total scores above or below the clinical threshold (in women defined as a CSFQ-14 total score ≤ 41, in men ≤47).*
