# Supplementary material for: Sexual health and serotonin 4 receptor brain binding in unmedicated patients with depression—a NeuroPharm study
Source: Transl Psychiatry. 2023 Jul 6;13:247. doi: 10.1038/s41398-023-02551-x (PMC10325956; doi:10.1038/s41398-023-02551-x)
Supplement: Supplementary file 2 — Supplementary figure 1 [file 41398_2023_2551_MOESM2_ESM.pptx]

## Slide 1
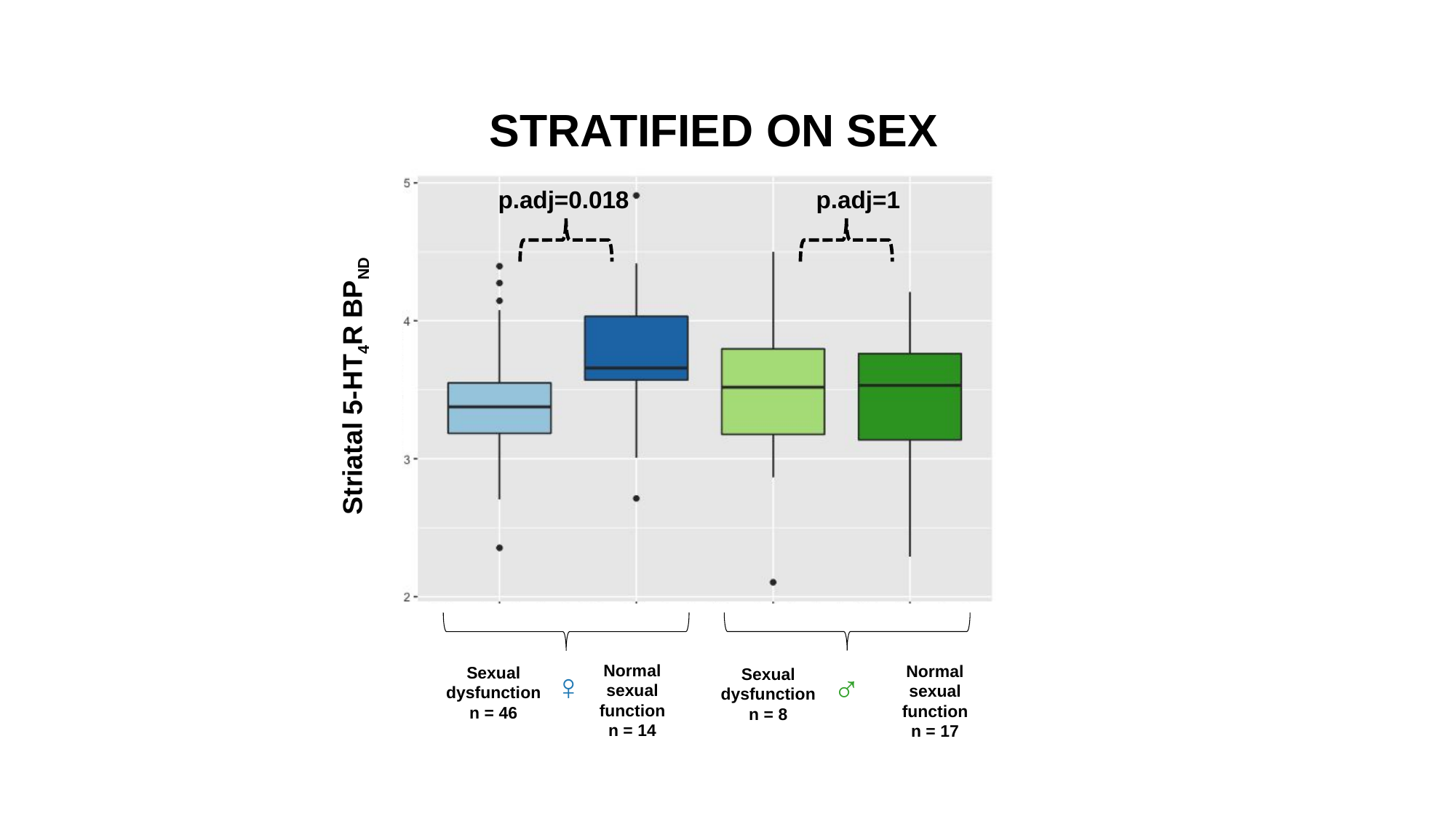

STRATIFIED ON SEX
p.adj=0.018
p.adj=1
Striatal 5-HT4R BPND
Normal sexual function
n = 14
Normal sexual function
n = 17
Sexual dysfunction
n = 46
♀
Sexual dysfunction
n = 8
♂
